# Association between postnatal anthropometric growth by term and high-performing neurodevelopment at age 3 years in extremely preterm infants

**DOI:** 10.1038/s41372-026-02692-z

**Published:** 2026-05-13

**Authors:** Takashi Maeda, Yoshihiro Tanahashi, Hideyuki Asada, Kazuto Ueda, Akinobu Taniguchi, Ryuichi Tanaka, Ryosuke Miura, Toshihiko Suzuki, Yukako Muramatsu, Eiko Kato, Seiji Hayashi, Hikaru Yamamoto, Koji Takemoto, Yuichi Kato, Makoto Oshiro, Hiroyuki Kidokoro, Yoshiyuki Takahashi, Yoshiaki Sato

**Affiliations:** 1 Division of Neonatology, Center for Maternal-Neonatal Care, Nagoya University Hospital, Nagoya, Japan; 2 Department of Pediatrics, Nagoya University Graduate School of Medicine, Nagoya, Japan; 3 Department of Pediatrics, Anjo Kosei Hospital, Anjo, Japan; 4 Department of Pediatrics, TOYOTA Memorial Hospital, Toyota, Japan; 5 Department of Neonatology, Ogaki Municipal Hospital, Ogaki, Japan; 6 Department of Pediatrics, Japanese Red Cross Aichi Medical Center Nagoya Daiichi Hospital, Nagoya, Japan; 7 Department of Pediatrics, Tosei General Hospital, Seto, Japan; 8 Department of Pediatrics, Okazaki City Hospital, Okazaki, Japan; 9 Department of Pediatrics, Konan Kosei Hospital, Konan, Japan

**Keywords:** Outcomes research, Organogenesis

## Abstract

**Objective:**

To evaluate the relationship between postnatal growth by term and high-performing neurodevelopment in infants born at less than 28 weeks of gestational age.

**Methods:**

This retrospective study examined associations between anthropometric Z-scores (length, weight, head circumference) at 32, 36, and 40 weeks postmenstrual age (PMA) and neurodevelopment at age 3 categorized into four groups (delay, subnormal, normal, high-performing).

**Results:**

A total of 287 infants were evaluated. Body length at 40 weeks PMA was significantly associated with high-performing neurodevelopment (adjusted OR = 1.86, 95% CI = 1.49–2.32), followed by head circumference (adjusted OR = 1.32, 95% CI = 1.04-67). Changes in body length Z-scores from 36 to 40 weeks PMA were significantly associated with high-performing neurodevelopment, whereas body weight and earlier changes in Z-scores were not.

**Conclusions:**

In extremely preterm infants, body length growth by 40 weeks PMA was an independent predictor for high-performing neurodevelopment at age 3, whereas body weight and earlier growth changes did not.

## Introduction

In recent decades, advances in perinatal care and medicine for extremely preterm infants have progressed dramatically, leading to a significant increase in the survival rates of this vulnerable population [1–3]. Despite this improved survival, a large number of extremely preterm infants (EPI) have displayed long-term disabilities, including cognitive, motor, behavioral, educational, emotional, and social challenges [4–6]. Even when brain lesions are not apparent on neuroimaging, EPI has exhibited a high rate of various neurodevelopmental disabilities, and the pathophysiological mechanisms of these problems are not well understood [7]. Clarifying the pathogenesis of its morbidity requires a multifaceted approach, particularly focusing on aspects related to neurodevelopment [7, 8]. Among the various contributing factors to this morbidity, such as sex, prematurity, and perinatal complications [9, 10], differences in intra-uterine and extra-uterine nutrition are considered important factors affecting the distinct brain development observed between term and preterm infants [10]. Previous studies investigating the association between slower growth and neurodevelopmental outcomes have mainly focused on postnatal growth in body weight and head circumference, both of which are associated with neurodevelopmental outcomes [11–13]. More recently, body length has also been highlighted as an important growth indicator [14, 15]. In addition, longitudinal assessments, such as changes in Z-scores, have been reported to better predict neurodevelopment than single cross-sectional measurements [16, 17]. Despite these insights, most previous research has focused on identifying risk factors for moderate or severe neurodevelopmental impairment.

In Japan, the Revision of the Kyoto Scale of Psychological Development-2001 (KSPD) [18] has been commonly used to assess the neurodevelopment of preterm infants, including in follow-up studies conducted by the Japan Neonatal Research Network [19, 20]. The KSPD correlates closely with the Bayley Scales of Infant and Toddler Development, 3rd edition, for assessing neurodevelopment in preterm infants [21]. It provides developmental age for both total and subscales, from which the developmental quotient (DQ) is derived using chronological age [18]. In our previous study, we suggested that setting a higher threshold for total DQ at age 3 years (≥93) better predicted freedom from educational support at school age than conventional cutoffs [22]. This concept of “high-performing neurodevelopment” enables the identification of children who are free from subtle impairments that may affect later academic and social functioning. Although children within the conventionally defined “normal” DQ range may appear developmentally typical in early childhood, our previous work demonstrated that a substantial proportion of them required educational support at school age [21]. This indicates that the traditional normal range does not necessarily guarantee favorable long-term functional outcomes. Therefore, distinguishing a subgroup with DQ ≥ 93 is clinically meaningful, as it identifies children with a more consistently favorable developmental trajectory, without implying that children in the normal range are inferior.

In this study, we incorporated the high-performing neurodevelopment at age 3 years [22], which we observed as the primary outcome. We collected nutritional data during the acute and subacute phases after birth in EPI and tracked their anthropometric Z-scores for body length, weight, and head circumference up to term-equivalent age using the Japanese neonatal anthropometric chart [23]. Our aim was to determine which anthropometric indicators and growth patterns were most significantly associated with high-performing neurodevelopment.

## Methods

### Subjects

**Fig. 1:**
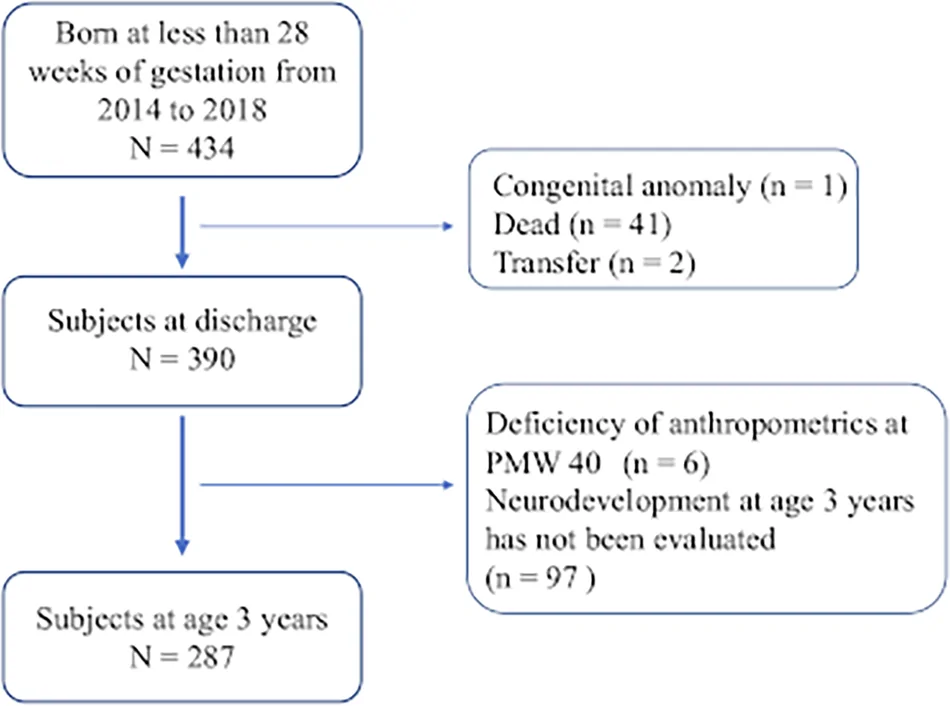
Flow diagram of study participants. A flow diagram illustrating the selection process of study participants from an initial cohort of 434 extremely preterm infants, leading to the final inclusion of 287 subjects.

The study included EPI born at less than 28 weeks of gestational age who were admitted between January 2014 and December 2018 to eight NICUs: Nagoya University Hospital, Anjo Kosei Hospital, Japanese Red Cross Aichi Medical Center, Nagoya Daiichi Hospital, Ogaki Municipal Hospital, Tosei General Hospital, Okazaki City Hospital, TOYOTA Memorial Hospital, and Konan Kosei Hospital. Eligible infants were those who survived hospitalization, had anthropometric measurements at 40 weeks of post menstrual age (PMA), and performed neurodevelopmental assessments at 3 years of age. Infants who were transferred to another hospital, those died during hospitalization, and those with significant congenital anomalies were excluded. Consequently, 287 infants were ultimately enrolled in this retrospective cohort study (Fig. 1).

### Anthropometrics measurement

Anthropometrics measurements of each infant during hospitalization were extracted from the medical records. Body weight was measured daily using an electronic scale before feeding and after diaper changes, with the infant unclothed. Body length and head circumference at predetermined times for each facility using non-stretch measuring tapes. We obtained the anthropometric data at 32-, 36-, and 40 weeks PMA. Z-scores for each measurement were derived based on Japanese neonatal anthropometric charts [23], and changes in Z-scores were also computed for the following intervals: from birth to 32 weeks, from 32 to 36 weeks, and from 36 to 40 weeks PMA.

### Data collection

The clinical characteristics including gestational age, sex, multiple births, hypertensive disorders of pregnancy, gestational diabetes mellitus, premature rupture of the membranes, clinical/histological chorioamnionitis, Apgar score, invasive ventilation, bronchopulmonary dysplasia (defined as the need for supplemental oxygen or respiratory support at 36 weeks PMA), grade III − IV of intraventricular hemorrhage (defined according to the Papile classification), periventricular leukomalacia (defined as the presence of cystic lesions on cranial ultrasound or MRI), blood culture-proven neonatal sepsis, focal intestinal perforation, necrotizing enterocolitis (defined as stage II or higher according to the Bell staging criteria), were collected. For enteral nutrition, data on the type (formula milk or breast milk) and amount of enteral feeding, day of initiation, days to achieve full feeding (100 ml/kg/day), and days of initiation and termination with fortified nutrition were recorded. For parenteral nutrition, the day of initiation and termination, duration, and the energy (kcal/kg/day), amino acid (g/kg/day), and fat (g/kg/day) intakes during the first two weeks after birth were collected.

### Neurodevelopmental assessment

Neurodevelopment at age 3 years in chronological age was assessed by certified phycologists, using the KSPD [18]. The total DQ was calculated based on three composite subscales: postural-motor, cognitive-adaptive and language-social. Based on our previous study, the participants were subdivided into four groups according to their neurodevelopmental outcomes: total DQ < 70 was classified as developmental delay, 70 ≤ total DQ < 85 as mild developmental delay, 85 ≤ total DQ < 93 as normal development, and total DQ ≥ 93 as high-performing [22].

### Statistical analysis

The categorical variables were given as numbers (percentages) and compared using the Chi-squared or Fisher’s exact test. The normal distributed quantitative variables were expressed as mean ± standard deviation (SD) and compared using the Students’ t-test or one-way ANOVA test. Where the overall one-way ANOVA test was statistically significant, post-hoc pairwise comparisons between the four neurodevelopmental groups were conducted using the Bonferroni -adjusted *p*-values to account for multiple comparisons. The non-normal distributed quantitative variables were expressed as the median (interquartile range) and compared using the Mann-Whitney U test or Kruskal-Wallis test. An adjusted model using logistic regression analysis was conducted to determine the association between anthropometric Z-scores during the neonatal period and high-performing neurodevelopment at age 3 years. Potential confounding factors for inclusion in the adjusted model were identified as variables that showed a statistically significant association (*p* < 0.05) with the outcome in univariate analyses, in addition to other clinically relevant potential confounders. In these models, sex was coded as a binary variable (male = 1, female = 0), with male sex coded as the risk category. In addition to analyzing Z-scores of anthropometric measurements at 32, 36, and 40 weeks of PMA, we also calculated the changes in Z-scores between time points (Birth-32 weeks, 32–36 weeks, and 36–40 weeks) for body weight, body length, and head circumference. These changes in Z-scores were included as explanatory variables in logistic regression models to evaluate their associations with high-performing neurodevelopment at age 3 years. In these logistic regression models, all anthropometric Z-scores and their changes over time were treated as continuous variables. To examine the association between institution and high-performing neurodevelopment, we performed logistic regression analysis, including potential factors as covariates to adjust for confounding. Adjusted receiver operating characteristic (ROC) curve analyses were performed based on logistic regression models. These models were adjusted for potential confounding factors identified in univariate analyses and clinically relevant variables. We assessed the diagnostic values for two distinct neurodevelopmental outcomes: (1) high-performing and (2) favorable neurodevelopment (combining the “normal” and “high-performing”). The optimal cut-off value was identified for each ROC curve using the Youden index (maximum sum of sensitivity and specificity). The association between variables related to nutritional methods and anthropometrics was evaluated using Spearman’s rank correlation coefficient. All comparisons were two-tailed, and a *p*-value < 0.05 was considered statistically significant. Statistical analysis was performed with SPSS statistical software package, version 28.0.

### Informed consent and ethical approval

Written consent was not obtained in this retrospective study with ethical approval. However, the opportunity for participants to decline participation was ensured by publicly disclosing information documents on each facility’s website, allowing for opt-out. This study was approved by the local ethical committee at Nagoya University Hospital (No. 2022-0274, approved 24 October 2022).

## Result

### Characteristics of patients

Table 1 summarizes the clinical characteristics of the 287 infants, presented for the total cohort and stratified by neurodevelopmental outcome at 3 years of age. The mean (SD) gestational age and birth weight of the cohort were 26.0 (1.4) weeks and 777 (210) grams, respectively. Gestational age and birth weight, as well as the incidences of intraventricular hemorrhage and bronchopulmonary dysplasia, differed significantly among groups. Anthropometric measurements at 36 and 40 weeks PMA are also presented.

**Table 1:** Clinical characteristics and anthropometric measurements of the study cohort, stratified by neurodevelopmental outcomes at age 3 years.

	Total (*n* = 287)	Developmental delay (*n* = 78)	Subnormal (*n* = 83)	Normal (*n* = 59)	High-performing (*n* = 67)	*p*-value^g^
Demographics						
Maternal age, *y*, mean (SD)	32.7 (5.1)	33.9 (4.8)	32.4 (5.4)	31.8 (5.2)	32.5 (5.0)	*0.69*
Chorioamnionitis, *n* [%]	137 [47.7]	37 [47.0]	42 [50.6]	28 [47.4]	30 [44.7]	*0.67*
Antenatal steroid, *n* [%]	142 [49.4]	29 [37.1]	34 [40.9]	45 [76.2]^e, f^	34 [50]	*< 0.01*
Apgar score1, mean (SD)	3.3 (2.0)	3.2 (1.9)	3.4 (2.1)	3.2 (1.7)	3.4 (2.1)	*0.72*
Apgar score5, mean (SD)	5.6 (2.0)	5.6 (2.1)	5.7 (2.1)	5.5 (1.9)	5.6 (2.1)	*0.83*
Caesarean section, *n* [%]	234 [81.5]	64 [82.0]	67 [80.7]	45 [76.2]	58 [86.5]	*0.52*
Male, *n* [%]	142 [49.4]	50 [64.1]	36 [43.3]	29 [49.1]	27 [40.2]^e^	*0.02*
Singlton, *n* [%]	222 [77.3]	60 [76.9]	66 [79.5]	44 [74.5]	52 [77.6]	*0.18*
Gestational age, week, mean (SD)	26 (1.4)	25.8 1.4)	25.7 (1.5)	26.3 (1.4)	26.4 (1.3)^e, f^	*< 0.01*
Birth weight, g, mean (SD)	777 (210)	692 (194)	767 198)	835 (220)^e^	835 (202)^e^	*< 0.01*
Birth weight Z-score, mean (SD)	−0.66 (1.6)	−1.20 (1.6)	−0.58 (1.1)	−0.23 (2.6)^e^	−0.53 (1.2)	*< 0.01*
Small for gestational age, *n* [%]	46 [16.0]	21 [26.9]	10 [12.0]	7 [11.8]	8 [11.9]	*0.02*
Neonatal morbidities						
Intraventricular hemorrhage, grade Ⅰor Ⅱ, *n* [%]^*a^	65 [22.6]	26 [33.3]	19 [22.8]	13 [22.0]	7 [10.4]^e^	*0.01*
Intraventricular hemorrhage, grade Ⅲ or Ⅳ, *n* [%]^*a^	16 [5.5]	8 [10.2]	6 [7.2]	2 [3.3]	0 [0.0]^e^	*0.01*
Cystic periventricular leukomalacia, *n* [%]^*b^	3 [1.0]	0 [0.0]	0 [0.0]	2 [3.3]	1 [1.4]	*0.14*
Ligation patent ductus arteriosus, *n* [%]	22 [7.7]	7 [8.9]	11 [13.2]	2 [3.3]	2 [2.9]	*0.06*
Blood culture-proven sepsis, *n* [%]	23 [8.0]	10 [12.8]	5 [6.0]	3 [5.1]	5 [7.4]	*0.33*
Necrotizing enterocolitis, n [%]	10 [3.5]	4 [5.1]	3 [3.6]	2 [3.6]	1 [1.5]	*0.70*
Bronchopulmonary dysplasia, *n* [%]^*c^	165 [57.4]	51 [65.3]	56 [67.4]	28 [47.4]	30 [44.7]^f^	*< 0.01*
Retinopathy of prematurity, *n* [%]^*d^	96 [33.4]	32 [41.0]	28 [33.7]	21 [35.5]	15 [22.3]	*0.12*
Anthropometrics measurement						
Body weight at 36wk of PMA, g, mean (SD)	1811 (285)	1612 (448)	1845 (317)^e^	1917 (400)^e^	1906 (357)^e^	*< 0.01*
Body length at 36wk of PMA, cm, mean (SD)	40.1 (3.2)	37.5 (2.7)	40.4 (2.8)^e^	41.5 (2.9)^e^	41.5 (2.8)^e^	*< 0.01*
Head circumstance at 36wk of PMA, cm, mean (SD)	30 (2.2)	29.2 (1.8)	30.1 (2.0)^e^	30.2 (2.0)^e^	30.5 (2.7)^e^	*< 0.01*
Body weight at 40wk of PMA, g, mean (SD)	2532 (511)	2300 (539)	2561 (423)^e^	2650 (531)^e^	2661 (478)^e^	*< 0.01*
Body length at 40wk of PMA, cm, mean (SD)	43.7 (3.7)	40.3 (2.6)	42.7 (2.5)^e^	45.9 (2.6)^e, f^	46.9 (2.8)^e, f^	*< 0.01*
Head circumstance at 40wk of PMA, cm, mean (SD)	32.9 (2.1)	32.1 (2.6)	32.8 (1.4)	33.6 (2.6)^e^	33.5 (2.0)^e^	*< 0.01*

*SD* standard deviation.

^*a^ Papile classification.

^*b^ Cystic lesion measuring larger than 1 mm identified on MRI.

^*c^ Requiring oxygen or respiratory support at 36 weeks of postmenstrual age.

^*d^ Requiring treatment during the neonatal period.

^e^*p* < 0.05 vs Developmental Delay.

^f^*p* < 0.05 vs Subnormal.

^g^*p*-value from one-way ANOVA for continuous variables and from chi-square test for categorical variables among the four groups.

### Neurodevelopment at age 3 years

Based on neurodevelopment classification, 78 children (27.1%) were subdivided as developmental delay, 83 children (28.9%) as subnormal, 59 children (20.5%) as normal development, and 67 children (23.3%) as high-performing [22]. The median (interquartile range) subscales were 89 (65–102) for Posture-Motor, 83 (69–94) for Cognitive-Adaptive, and 81 (65–92) for Language-Social. Supplementary Table 1 shows the neurodevelopment at age 3 years using the KSPD. In addition, Table 1 presents comparisons of clinical characteristics among the four neurodevelopmental groups (developmental delay [*n* = 78], subnormal [*n* = 83], normal [*n* = 59], and high-performing [*n* = 67]). Infants in the high-performing group had significantly higher gestational age and birth weight, and lower incidence of severe intraventricular hemorrhage (grade III–IV) and bronchopulmonary dysplasia.

### Growth patterns of anthropometrics in each neurodevelopment group

**Fig. 2:**
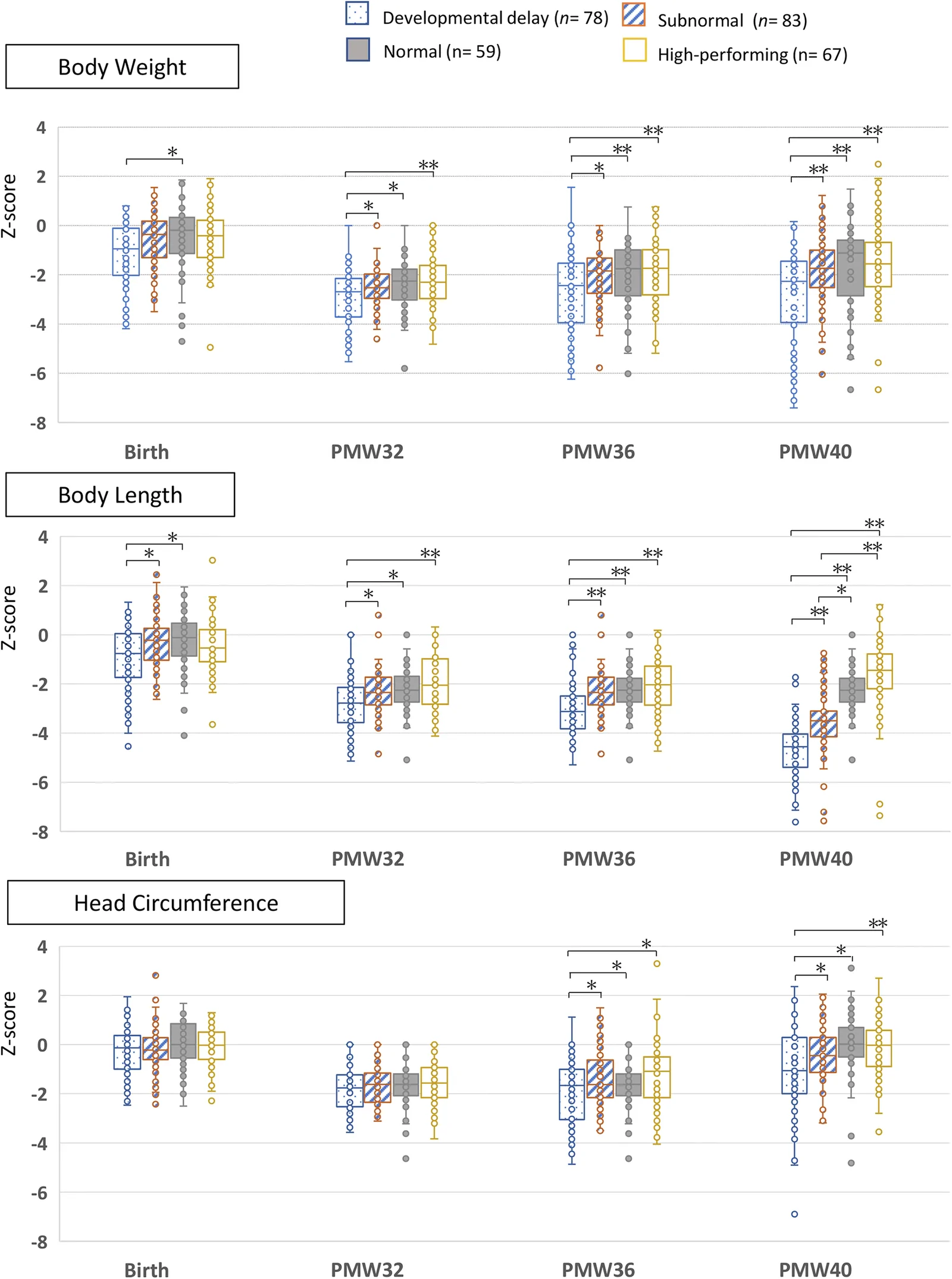
Comparison of crude growth patterns of anthropometric Z-scores across neurodevelopmental groups. The crude Z-scores for body weight, body length, and head circumference are shown for the four neurodevelopmental groups: developmental delay (*n* = 78), subnormal (*n* = 83), normal (*n* = 59), and high-performing neurodevelopment (*n* = 67). Statistically significant differences derived from post-hoc pairwise comparisons are indicated (* indicates *p*_*adj*_ < 0.05, and ** indicates adjusted *p*_*adj*_ < 0.01. PMW post menstrual week.

Figure 2 shows the unadjusted growth patterns of Z-scores for body weight, body length, and head circumference, analyzed across the four neurodevelopmental groups. Comparisons between the four groups at each time point were made using one-way ANOVA, followed by post-hoc tests with Bonferroni correction where appropriate. By 32 weeks PMA, the developmental delay group already showed signs of delayed growth in body weight and length. At 36 weeks PMA and 40 weeks PMA, the subnormal, normal, and high-performing groups demonstrated significantly better catch-up growth in all growth indicators compared to the developmental delay group. Particularly at 40 weeks PMA, the differences in body length between the neurodevelopmental groups were the most pronounced, with the subnormal (*p* < 0.001), normal (*p* = 0.001), and high-performing (*p* < 0.001) groups showing significantly better growth compared to the developmental delay group. Moreover, significant differences in growth were also observed between the subnormal and normal groups (*p* < 0.001), as well as between the subnormal and high-performing groups (*p* < 0.001). It is important to note, however, that despite these significant differences in mean Z-scores, there was considerable overlap in the distribution of anthropometric values among the four groups at each time point.

### Neurodevelopment and anthropometrics

Table 2 shows the results of the multiple logistic regression analyses, which present two distinct models. The first model, for the ‘Entire cohort,’ evaluates the association between anthropometric measurements and achieving high-performing neurodevelopment compared to a combined reference group of all other participants. The second model presents a subgroup analysis restricted to infants with normal or high-performing neurodevelopment. A logistic regression analysis, adjusted for confounding factors including gestational age, birth weight, sex, intraventricular hemorrhage, bronchopulmonary dysplasia, and small for gestational age, revealed that body length Z-score at 36 weeks PMA (*p*-value = 0.02, OR = 1.49, 95%CI = 1.08–2.06), body length Z-score at 40 weeks PMA (*p*-value < 0.001, OR = 1.86, 95%CI = 1.49–2.32), and head circumference Z-scores at 40 weeks PMA (*p*-value = 0.02, OR = 1.32, 95%CI = 1.04–1.67) were significant variables for the high-performing neurodevelopment. In the subgroup analysis between the normal and high-performing, body length at 40 weeks PMA was the only anthropometric measure that showed a significant difference, being higher in the high-performing. Furthermore, the change in body length Z-score from 36 to 40 weeks PMA was also found to be significantly associated with high-performing neurodevelopment (OR = 1.98, 95% CI: 1.55–2.62, *p* = 0.001), whereas changes in weight or head circumference Z-scores during the same interval were not significant. Importantly, this association was also observed in the subgroup with normal and high-performing (OR = 1.70, 95% CI: 1.18–2.45, *p* = 0.004). Whereas changes in Z-scores from birth to 32 weeks PMA and from 32 to 36 weeks PMA were not significantly associated with outcome in any of the anthropometric measures.

**Table 2:** Anthropometric Z-scores, Z-scores changes and high-performing neurodevelopment at age 3 years.

		Entire cohort		Normal and high-performing	
		OR	95%CI	OR	95%CI
Anthropometrics Z score					
Birth	Body Weight	0.97	0.75–1.24	0.62	0.30–1.28
	Body Length	0.88	0.66–1.18	0.68	0.44–1.06
	Head Circumstance	0.98	0.77–1.24	0.93	0.49–1.79
PMW 32	Body Weight	1.49	0.95–2.34	1.53	0.81–2.88
	Body Length	1.25	0.81–1.94	1.10	0.61–1.96
	Head Circumstance	0.95	0.57–1.57	1.08	0.57–2.01
PMW 36	Body Weight	1.25	0.93–1.70	0.98	0.65–1.48
	Body Length	1.49	1.08–2.06	1.00	0.66–1.52
	Head Circumstance	1.22	0.91–1.63	0.87	0.87–1.83
PMW 40	Body Weight	1.25	0.99–1.58	1.00	0.75–1.33
	Body Length	1.86**	1.49–2.32	1.38*	1.04–1.85
	Head Circumstance	1.32*	1.04–1.67	1.04	0.78–1.38
Anthropometric Z-score changes					
Body Weight	Birth–PMW 32	1.44	0.95–2.20	1.62	0.91–2.82
	PMW 32–PMW 36	0.94	0.63–1.41	0.78	0.46–1.32
	PMW 36–PMW 40	1.20	0.82–1.75	1.08	0.65–1.81
Body Length	Birth–PMW 32	1.30	0.90–1.88	1.49	0.91–2.44
	PMW 32–PMW 36	1.30	0.94–1.78	1.00	0.66–1.51
	PMW 36–PMW 40	1.98**	1.55–2.62	1.70**	1.18–2.45
Head Circumstance	Birth–PMW 32	0.98	0.65–1.50	1.22	0.71–2.11
	PMW 32–PMW 36	1.21	0.87–1.70	1.11	0.74–1.67
	PMW 36–PMW 40	1.17	0.91–1.57	0.88	0.60–1.29

Multivariate models were adjusted for potential confounders, including gestational age, birth weight, sex, grade III–IV intraventricular hemorrhage, bronchopulmonary dysplasia, and small for gestational age.

*OR* odds ratio, *CI* confidence interval, *PMW* postmenstrual week.

*p < 0.05; **p < 0.01.

**Fig. 3:**
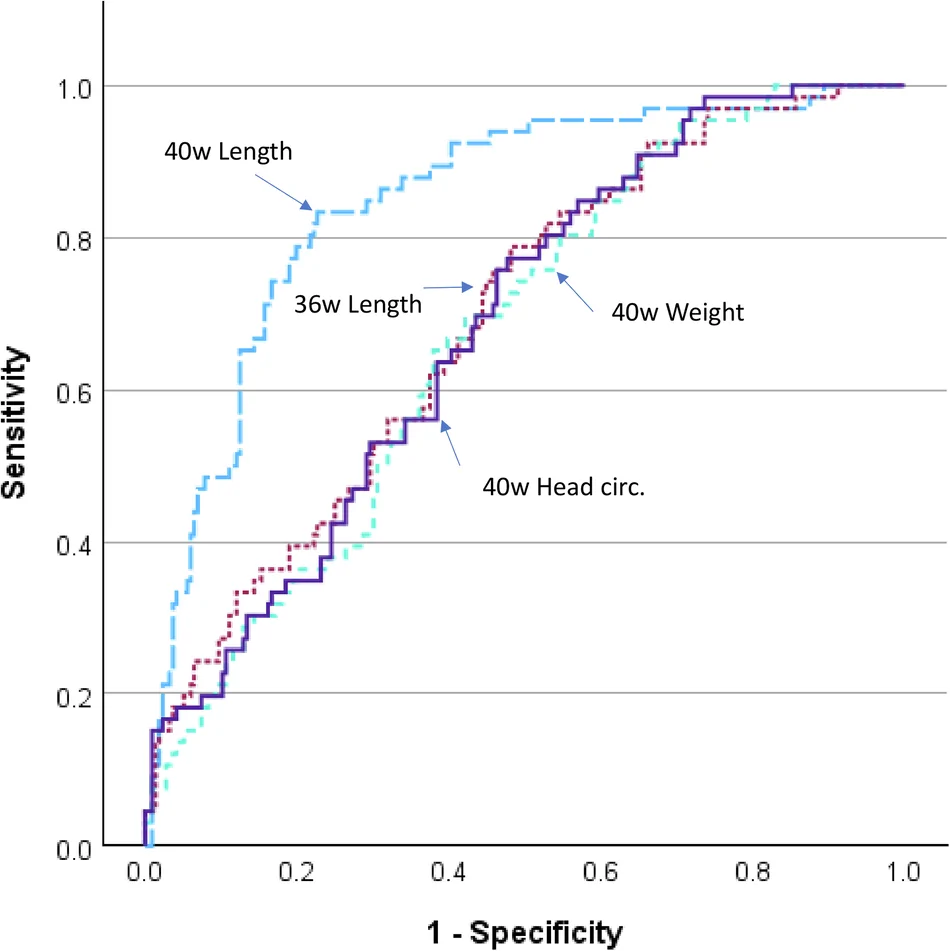
Adjusted Receiver operating characteristic (ROC) curves for predicting high-performing neurodevelopment within the entire cohort. Adjusted ROC curves illustrating the predictive ability of anthropometric Z-scores (body length at 36 weeks and 40 weeks PMA, body weight and head circumference at 40 weeks PMA) for high-performing neurodevelopment at 3 years of age, analyzed within the entire cohort. 40w Length, body length at 40 weeks; 36w Length, body length at 36 weeks; 40w Weight, body weight at 40 weeks; 40w Head circ, head circumstance at 40 weeks.

Supplementary Table 2 shows the results of logistic regression analyses examining the association between institution and high-performing neurodevelopment, and the associations between anthropometric Z-scores, their changes, and high-performing neurodevelopment at age 3 years. Even after adjustment for potential confounders, including institution, body length at 40 weeks, and the change in body length from 36 to 40 weeks, PMA remained significantly associated with high-performing neurodevelopment. In the analysis within the entire cohort, the areas under the adjusted ROC curves for predicting high-performing neurodevelopment were 0.851 (95% CI: 0.801–0.901) for body length Z-score at 40 weeks PMA, 0.700 (95% CI: 0.631–0.768) for body length Z-score at 36 weeks PMA, and 0.677 (95% CI: 0.610–0.745) for head circumference Z-score at 40 weeks PMA (Fig. 3). Additionally, with −2.93 of Z-score as a cut-off value, the sensitivity of the high-performing neurodevelopment was 69% and specificity was 89%. Furthermore, an adjusted ROC analysis was conducted for favorable neurodevelopment (combining normal and high-performing). The AUC for body length Z-score at 40 weeks PMA was 0.897 (95% CI: 0.858–0.936) (Supplementary Fig.).

### Nutrition factors and anthropometrics

Supplementary Table 3 shows the correlation between anthropometrics measurements and nutritional factors during the acute (first two weeks of life) and subacute (3–4 weeks of life) postnatal period. The initiation and termination day of age with parenteral nutrition, the day of age which enteral nutrition reached 100 ml/kg/day, and the day of age at which fortified nutrition was started were negatively correlated body length Z-score at 40 weeks PMA, while energy intake up to 2 weeks and 2–4 weeks from birth were positively correlated with body length Z-score at 40 weeks PMA, and these correlations were also observed for body weight Z-score at 40 weeks PMA and head circumference Z-score at 40 weeks PMA.

## Discussion

In this multicenter retrospective study of EPI, we investigated the relationship between the growth of anthropometric Z-scores by term and neurodevelopmental outcomes at age 3 years. Compared to head circumference and body weight, linear growth lagged, with many infants remaining below the expected range for body length at term-equivalent age. Notably, infants who achieved high-performing neurodevelopment demonstrated significantly greater linear growth, and body length at 40 weeks PMA significantly predict for high-performing neurodevelopment. Furthermore, in a subgroup analysis restricted to infants with normal or high-performing neurodevelopment, only body length at term was independently associated with high-performing neurodevelopment among all anthropometrics.

Our longitudinal results on anthropometrics at term were consistent with previous studies on EPI [24–26]. Horemuzova et al. reported mean Z-scores at 40 weeks PMA for weight, length, and head circumference as −1.72, −2.49, and −0.86, respectively, with about 70% of infants having a length Z-score below −2 SD at discharge [24]. This imbalance in catch-up growth suggests the need for improved nutritional interventions for postnatal extrauterine growth restriction. Insufficient growth in weight and head circumference during the NICU stay is a well-established risk factor for neurodevelopmental impairment [12, 13, 16, 27–30]. However, most reports focused on moderate or severe neurodevelopmental disabilities [13, 16, 29, 30], while many EPI exhibit milder impairments like attention deficits, learning difficulties, and mild motor delays [4–6], which require further discussion regarding their pathophysiology. In our previous study, we demonstrated that setting a higher threshold for total DQ at age 3 years significantly reduced the number of children who could attend regular classes without educational support [22]. Based on these findings, we have proposed a new neurodevelopmental benchmark at age 3 years that should be targeted in the future [22].

In this study, the high-performing neurodevelopment group showed better growth in body length compared to others. Additionally, subgroup analysis comparing the normal and high-performing neurodevelopment groups revealed that body length at 40 weeks PMA was the only anthropometric measure with a significant difference. This association between high-performing neurodevelopment and body length offers valuable insights for improving perinatal care to enhance neurodevelopmental outcomes in EPI. Our findings on the association between high-performing neurodevelopment and body length suggested the importance of the quality of postnatal nutrition and balancing energy intake and expenditure for achieving high-performing neurodevelopment. Recent studies have increasingly focused on growth indicators other than weight and head circumference. Fat-free mass at term has been associated with brain volume, serving as a biomarker of postnatal nutrition [31]. Studies have also shown that postnatal body length growth correlates with neurodevelopment in preterm infants. Watanabe et al. found body length and head circumference at term were key determinants of white matter volume [15], Egashira et al. linked them to cognitive function and DQ > 85 at age 3 in very low birth weight infants [14]. In our population, when using higher threshold for high-performing neurodevelopment, height emerged as the most reliable indicator. Head circumference growth is often preserved by the ‘brain sparing effect’ during nutritional deficiency [32]. Additionally, in the NICU, nutritional interventions have focused on weight gain, leading to fat accumulation [33–35], while essential nutrients for bone and muscle growth may be insufficient, leading to result in delayed growth of body length [34]. While weight gain occurs promptly, growth of body length requires long-term, stable nutrition [34, 36, 37]. Thus, body length may be a more sensitive indicator of postnatal nutritional status [38]. Our finding that the change in body length Z-score from 36 to 40 weeks was most strongly associated with high-performing neurodevelopment supports this notion. We hypothesize that this 36–40 weeks period reflects a critical transitional phase in preterm physiology. We speculate that during this time, many infants recover from the catabolic stress of acute complications (like bronchopulmonary dysplasia or infection), allowing stable nutrition to be efficiently allocated beyond fat accretion toward quality linear growth to begin catch up growth towards their genetic potential. Furthermore, we propose that the body length Z-score at 40 weeks itself might be an indicator of an infant’s feeding ability [39]. The NICU course is frequently challenged by complications that can slow growth. Therefore, body length at 40 weeks PMA may reliably reflect which some infants successfully begin catch-up growth.

We investigated the relationship between postnatal nutritional factors—energy intake, days to full enteral nutrition, and duration of total parenteral nutrition—and anthropometric measurements. These factors were significantly associated with growth in body length, head circumference, and weight, but none were specifically linked to body length in term infants. Linear growth requires nutrients like vitamin D, calcium, phosphorus, and hormones such as growth hormone and insulin-like growth factor-1 [40, 41]. A deficiency in these nutrients may result in delayed linear growth, and particularly in EPI, the immaturity of the secretion and action of these hormones leads to further delays [42]. Complications like respiratory distress and infections can also increase energy needs, reducing resources for growth [43]. Taken together, growth should be regarded as an intermediate variable influenced by nutritional, hormonal, and medical factors, and identifying these determinants is essential for developing more effective nutritional and medical strategies to improve long-term neurodevelopmental outcomes.

This study has several limitations. First, we were unable to consider the social and educational backgrounds of the subjects due to the lack of family information. These factors may affect the cognitive and language abilities of infants [44]. Second, the nutritional management practices in the NICU varied across centers, making it difficult to standardize interventions. Additionally, anthropometric measurements in this study were obtained using non-stretchable tape measures, which may be less accurate than more advanced techniques such as length boards or imaging-based assessments, particularly in critically ill infants with severe morbidities. Moreover, infants without physical measurements at 40 weeks PMA or neurodevelopmental assessments at 3 years of age were excluded from the study. This exclusion could introduce selection bias, as infants lost to follow-up may differ systematically from those assessed, potentially having worse neurodevelopmental outcomes or facing greater socioeconomic challenges, which are themselves risk factors for poorer development. Furthermore, long-term follow-up beyond 3 years was not conducted, limiting our understanding of the long-term impact of early growth on neurodevelopment. Lastly, given the number of statistical tests performed—particularly within the regression analyses—the possibility of type I error inflation cannot be completely excluded. Therefore, these findings should be interpreted with appropriate caution, especially in the context of exploratory analyses.

In conclusion, our study highlights a significant association between body length growth by term and high-performing neurodevelopment at age 3 years in EPI. Body length may provide a more sensitive indicator of postnatal nutrition. Further research is needed to explore additional factors influencing height growth and to develop better nutritional interventions to enhance outcomes in EPI.

## Supplementary information

Supplemental Table 1

Supplemental Table 2

Supplemental Table 3

Supplemental Figure

Supplemental Figure Legends

## Data Availability

The datasets generated and/or analyzed during the current study are not publicly available due to ethical and privacy restrictions, but are available from the corresponding author on reasonable request and with appropriate institutional approval.
