# Supplementary material for: Association between postnatal anthropometric growth by term and high-performing neurodevelopment at age 3 years in extremely preterm infants
Source: J Perinatol. 2026 May 13;46(9):1641–8. doi: 10.1038/s41372-026-02692-z (PMC13588745; doi:10.1038/s41372-026-02692-z)
Supplement: Supplementary file 1 [file 41372_2026_2692_MOESM1_ESM.docx]

**Supplemental Table 1.**

Neurodevelopment at age 3 years

| Total DQ, median (interquartile range) | 81 (67–92) |
| --- | --- |
| Developmental delay, n [%] | 78 [27.1] |
| Subnormal, n [%] | 83 [28.9] |
| Normal, n [%] | 59 [20.5] |
| Ideal development, n [%] | 67 [23.3] |
| PM, median (interquartile range) | 89 (65–102) |
| CA, median (interquartile range) | 83 (69–94) |
| LS, median (interquartile range) | 81 (65–92) |

DQ, developmental quotient; PM, posture-Motor; CA, Cognitive-Adaptive; LS, Language-Social

Text summary

At age 3 years, the neurodevelopmental status of the cohort was assessed using the developmental quotient (DQ), with the median total DQ being 81 (interquartile range: 67–92).

Subscale medians (with interquartile ranges) were: Posture-Motor (PM): 89 (65–102), Cognitive-Adaptive (CA): 83 (69–94), Language-Social (LS): 81 (65–92)
