# Supplementary material for: Association between postnatal anthropometric growth by term and high-performing neurodevelopment at age 3 years in extremely preterm infants
Source: J Perinatol. 2026 May 13;46(9):1641–8. doi: 10.1038/s41372-026-02692-z (PMC13588745; doi:10.1038/s41372-026-02692-z)
Supplement: Supplementary file 2 [file 41372_2026_2692_MOESM2_ESM.docx]

**Supplemental Table 2a.** Association Between Optimal Development and Institution: A Logistic Regression Analysis

|  | OR (95% CI) | p-value |
| --- | --- | --- |
| Institution A | 2.82 (0.51-15.4) | 0.23 |
| Institution B | 2.86 (0.48-17.0) | 0.24 |
| Institution C | 11.1 (1.49-82.8) | 0.01 |
| Institution D | 1.87 (0.26-13.6) | 0.53 |
| Institution E | 1.22 (0.22-6.62) | 0.81 |
| Institution F | 1.72 (0.22-13.5) | 0.60 |
| Institution G | 1.54 (0.10-23.6) | 0.75 |
| Institution H | 1.33 (0.47-3.41) | 0.47 |

OR, odds ratio; CI, confidence interval; PMW, postmenstrual week

Multivariate models were adjusted for potential confounders including gestational age, birth weight, sex, grade III–IV intraventricular hemorrhage, bronchopulmonary dysplasia, and small for gestational age.

**Supplemental Table 2b.** Anthropometric Z-scores, Z-scores changes and high-performing neurodevelopment at age 3 years

|  |  |  |  | Entire cohort | |  | Normal and optimal development | |
| --- | --- | --- | --- | --- | --- | --- | --- | --- |
|  |  |  |  | OR | 95%CI |  | OR | 95%CI |
| Anthropometrics Z score | | | | | | | | |
| Birth | Body Weight | |  | 0.93 | 0.62–1.39 |  | 0.64 | 0.21–1.94 |
|  | Body Length | |  | 0.78 | 0.51–1.18 |  | 0.68 | 0.41–1.15 |
|  | Head Circumstance | |  | 1.02 | 0.80–1.29 |  | 1.31 | 0.59–2.87 |
| PMW 32 | Body Weight | |  | 1.53 | 0.90–2.60 |  | 1.80 | 0.83–3.91 |
|  | Body Length | |  | 1.25 | 0.81–1.94 |  | 1.10 | 0.61–1.96 |
|  | Head Circumstance | |  | 1.04 | 0.60–1.81 |  | 1.11 | 0.53–2.29 |
| PMW 36 | Body Weight | |  | 1.18 | 0.84–1.68 |  | 1.12 | 0.69–1.84 |
|  | Body Length | |  | 1.36 | 0.95–1.96 |  | 0.82 | 0.48–1.37 |
|  | Head Circumstance | |  | 1.11 | 0.80–1.53 |  | 1.36 | 0.83–2.22 |
| PMW 40 | Body Weight | |  | 1.16 | 0.90–1.49 |  | 1.10 | 0.78–1.56 |
|  | Body Length | |  | **1.98**** | **1.54–2.56** |  | **1.50*** | **1.03–2.18** |
|  | Head Circumstance | |  | 1.18 | 0.91–1.53 |  | 0.98 | 0.69–1.39 |
| Anthropometric Z score changes | | | | | | | | |
| Body Weight | Birth–PMW 32 | |  | 1.36 | 0.88–2.08 |  | 1.69 | 0.90–3.16 |
|  | PMW 32–PMW 36 | |  | 0.94 | 0.60–1.47 |  | 0.81 | 0.44–1.47 |
|  | PMW 36–PMW 40 | |  | 1.18 | 0.78–1.77 |  | 1.11 | 0.63–1.96 |
| Body Length | Birth–PMW 32 | |  | 1.32 | 0.89–1.95 |  | 1.35 | 0.79–2.31 |
|  | PMW 32–PMW 36 | |  | 1.21 | 0.84–1.74 |  | 0.82 | 0.47–1.41 |
|  | PMW 36–PMW 40 | |  | **1.98**** | **1.50–2.61** |  | **1.87**** | **1.21–2.88** |
| Head Circumstance | Birth–PMW 32 | |  | 0.90 | 0.57–1.42 |  | 0.96 | 0.51–1.83 |
|  | PMW 32–PMW 36 | |  | 0.99 | 0.65–1.49 |  | 1.07 | 0.55–2.08 |
|  | PMW 36–PMW 40 | |  | 1.12 | 0.82–1.53 |  | 0.76 | 0.49–1.17 |

OR, odds ratio; CI, confidence interval; PMW, postmenstrual week

Multivariate models were adjusted for potential confounders including gestational age, birth weight, sex, grade III–IV intraventricular hemorrhage, bronchopulmonary dysplasia, small for gestational age, and institution. **p<0.05*; ***p<0.01*
