# Supplementary material for: Association between postnatal anthropometric growth by term and high-performing neurodevelopment at age 3 years in extremely preterm infants
Source: J Perinatol. 2026 May 13;46(9):1641–8. doi: 10.1038/s41372-026-02692-z (PMC13588745; doi:10.1038/s41372-026-02692-z)
Supplement: Supplementary file 3 [file 41372_2026_2692_MOESM3_ESM.docx]

**Supplemental Table 3.**

Correlation between nutritional intake and anthropometric measurements at 36 and 40 weeks of post menstrual age. Values are Pearson’s correlation coefficients (R).

|  | PMW 36 | | | PMW 40 | | |
| --- | --- | --- | --- | --- | --- | --- |
|  | BW | BL | HC | BW | BL | HC |
| TPN initiation day | -0.05 | **-0.16**** | -0.04 | -0.05 | **-0.16**** | 0.01 |
| TPN termination day | **-0.31**** | -0.09 | **-0.23**** | **0.35**** | **-0.18**** | **-0.24**** |
| Protein intake via TPN (–2 weeks) | -0.06 | -0.03 | -0.08 | -0.01 | -0.08 | -0.01 |
| Fat intake via TPN (–2 weeks) | 0.05 | 0.07 | 0.09 | 0.11 | -0.01 | 0.07 |
| Enteral feeding start day | **-0.12*** | **-0.26**** | -0.09 | -0.07 | -0.09 | -0.10 |
| Full feeding day | **-0.37**** | **-0.18**** | **-0.32**** | **-0.37**** | **-0.18**** | **-0.34**** |
| Fortified feeding start day | **-0.37**** | **-0.26**** | **-0.28**** | **-0.38**** | **-0.17**** | **-0.29**** |
| Energy intake (–2 weeks) kcal/kg/day | **0.28**** | **0.27**** | **0.25**** | **0.25**** | **0.26**** | **0.27**** |
| Energy intake (2–4 weeks) kcal/kg/day | **0.46**** | **0.28**** | **0.4**** | **0.3**** | **0.23**** | **0.35**** |

* p < 0.05, ** p < 0.01

BW, body weight; BL, body length; HC, head circumference; PMW, postmenstrual weeks; TPN, total parenteral nutrition.

Significant positive associations were observed between energy intake and growth parameters, while delayed enteral and fortified feeding initiation were negatively correlated with body weight, length, and head circumference.
