# Supplementary material for: Association between postnatal anthropometric growth by term and high-performing neurodevelopment at age 3 years in extremely preterm infants
Source: J Perinatol. 2026 May 13;46(9):1641–8. doi: 10.1038/s41372-026-02692-z (PMC13588745; doi:10.1038/s41372-026-02692-z)
Supplement: Supplementary file 4 [file 41372_2026_2692_MOESM5_ESM.docx]

**Supplemental Figure Legends**

**Supplemental Figure: Adjusted Receiver operating characteristic (ROC) curves for predicting favorable neurodevelopment within the entire cohort**

Adjusted ROC curves illustrating the predictive ability of anthropometric Z-scores (body length at 36 weeks and 40 weeks PMA, body weight and head circumference at 40 weeks PMA) for favorable neurodevelopment (combining normal and high-performing groups) at 3 years of age. The models were adjusted for potential confounding factors identified in univariate analyses and clinically relevant variables. 40w Length, body length at 40 weeks; 36w Length, body length at 36 weeks; 40w Weight, body weight at 40 weeks; 40w Head circ, head circumference at 40 weeks. PMA, postmenstrual age.
